# Multigenerational Epigenetic Regulation of Allergic Diseases: Utilizing an Experimental Dust Mite-Induced Asthma Model

**DOI:** 10.3389/fgene.2021.624561

**Published:** 2021-04-01

**Authors:** Jairus C. Pulczinski, Yan Shang, Tyna Dao, Nathachit Limjunyawong, Qinying Sun, Wayne Mitzner, Robert YS Cheng, Wan-yee Tang

**Affiliations:** ^1^Department of Environmental Health and Engineering, Johns Hopkins Bloomberg School of Public Health, Baltimore, MD, United States; ^2^Center for Cancer Research, National Cancer Institute, Frederick, MD, United States; ^3^Department of Environmental and Occupational Health, University of Pittsburgh Graduate School of Public Health, Pittsburgh, PA, United States

**Keywords:** asthma, epigenetic inheritance, allergen, DNA methylation, DNA hydroxymethylation

## Abstract

Environmental exposures have been linked to increased asthma risk, particularly during pregnancy and in early life. Here we use a mouse model of allergic lung disease to examine the effects of pre- and perinatal house dust mite (HDM) allergen exposure on offspring phenotypic and transcriptional outcomes in three generations. We show that maternal HDM exposure (F0) acts synergistically with adult HDM exposure, leading to enhanced airway hyperresponsiveness (AHR) and lung inflammation when compared to mice exposed solely in adulthood. Additionally, a subset of F1 males were not challenged in adulthood, and used to generate F2 progeny, which was then used to generate F3 progeny. Upon adult challenge to HDM, F2, and F3 males generated from the maternal HDM (F0) exposure lineage displayed increased airway reactivity and inflammation when compared to mice exposed solely in adulthood. These findings indicate that maternal allergen exposure is capable of enhancing either susceptibly to or severity of allergic airway disease. To examine the role of epigenetic inheritance of asthma susceptibility induced by maternal HDM exposure, we utilized a genome-wide MeDIP-seq and hMeDIP-seq analysis to identify genes differentially methylated (DMG) and hydroxymethylated (DHG), and their association with the enhanced AHR. In addition, we validated the relationship between DNA methylation and mRNA expression of the DMGs and DHGs in the male sub-generations (F1-F3). We found the expression of *Kchn1*, *Nron*, and *Spag17* to be differentially hydroxymethylated and upregulated in the F1 exposed to HDM both in early life and in adulthood when compared to F1 mice exposed solely in adulthood. *Kcnh1* remained upregulated in the F2 and F3 from the maternal HDM (F0) exposure lineage, when compared to F1 mice exposed solely in adulthood. In summary, we demonstrated that maternal HDM exposure in early life can alter the gene expression and phenotype of offspring upon adult HDM exposure, resulting in more severe disease. These effects persist at least two generations past the initial insult, transmitted along the paternal line.

## Introduction

Asthma is a common chronic disease, afflicting some 330 million individuals globally and resulting in substantial morbidity and mortality, particularly among children (Dharmage et al., 2019). Asthma rates have increased in the last few decades and it is estimated that global asthma cases will surpass 400 million in 2025 (Dharmage et al., 2019). In part due to this increase, considerable efforts have been undertaken to understand asthma risk factors and underlying mechanisms. As maternal asthma confers a greater risk to the offspring than paternal asthma, it is thought that offspring may be vulnerable to maternally mediated exposures in the pre- and perinatal period (Lim et al., 2010). Research has shown that a broad range of maternal exposures, in humans and in animal models, can increase offspring asthma susceptibility, including particulate matter, maternal stress, smoking, and allergens (Celedón et al., 2002; Lim et al., 2010; Van De Loo et al., 2016; Lee et al., 2018; Dharmage et al., 2019). DNA methylation has been proposed as one of the underlying mechanisms, given the associations between *in utero* exposures to asthma risk factors, including air pollution and cigarette smoke, and changes in global and gene specific DNA methylation of offspring (Perera et al., 2009; Herbstman et al., 2012; Lee et al., 2017). In many cases, these perturbations are in the regulatory regions of immune related genes, including *IFNG* and *FOXP3*, thus providing biological plausibility (Tang et al., 2012; Hew et al., 2015).

As our understanding of maternal exposures and their epigenetic ramifications grows stronger, new research has emerged indicating that certain exposures may have the capacity to result in multigenerational inheritance of allergic disease (Mørkve Knudsen et al., 2018). Multigenerational inheritance is a phenomenon where exposure to an insult or stimulus results in a phenotypic effect in the subsequent generation. This phenomenon can be further subdivided into intergenerational and transgenerational effects. Intergenerational effects are those seen when the offspring is directly exposed to an insult, including as gametes. In mammals, this includes the F1 generation after exposure to the F0, as the sperm or ovum of the F0 is also exposed to the insult. In the case of prenatal exposure, this can also include the F2 generation, as the germline cells of female F1 offspring are also present during the insult. This contrasts with transgenerational inheritance, in which the F3 generation, or F2 from *in utero* exposed males, display phenotypic variation due to exposure in the F0 (Mørkve Knudsen et al., 2018).

Multigenerational exposure is challenging to identify epidemiologically due to the long lifespan of humans and the ubiquitous nature of many exposures. The strongest human data on multigenerational exposure and allergic disease risk comes from cohort studies examining grandparent smoking on offspring allergic disease risk, in part because cigarette smoke exposure *in utero* is one of the strongest risk factors for asthma development. In several cohorts, grandmaternal, but not grandpaternal smoking was significantly associated with grandchild asthma risk. This was independent of maternal smoking, however, maternal smoking magnified the risk (Mørkve Knudsen et al., 2018). A separate study found that paternal grandparent smoking was associated with increased asthma risk in grandchildren. Several other studies have examined the risk of preconception smoking on asthma risk, and also found that paternal smoking prior to conception increased the F1 offspring risk of asthma and reduced offspring lung function (Mørkve Knudsen et al., 2018). Taken together, these studies offer the strongest case that asthma risk is in part influenced via multigenerational exposure. The synergistic relationship between grandmaternal and maternal exposure is particularly relevant to public health, as many of our exposures, like air pollution, cigarette smoke, endocrine disrupters, and maternal stress, are not limited to an older generation, but are persistent and ubiquitous. If such exposures are capable of multigenerational inheritance of airway hyperresponsiveness (AHR), there may be a synergistic effect due to exposure in the F0, F1, and F2.

Given the diverse stimuli capable of producing multigenerational inheritance of exposure, and work in our lab and others indicating that multigenerational epigenetic programming may be contributing to asthma risk, we sought to investigate this phenomenon from several approaches. Our lab utilized a previously validated model of mouse allergic airway disease (Shang et al., 2013; Cheng et al., 2014), in which mice are sensitized to house dust mite (HDM) allergen. HDM is an environmentally relevant and ubiquitous indoor allergen, which many asthmatics are sensitized to Gaffin and Phipatanakul (2009). The administration of HDM elicits a strong immune response, characterized by T-helper cell 2 (TH2) skewing of the immune system, and increased AHR, similar to the TH2 high atopic asthma endotype (Kuruvilla et al., 2019). Our goal was to identify the contribution of maternal HDM exposure on offspring allergic airway disease risk and severity, and test our hypothesis that the inheritance of lung phenotypic changes is modulated by epigenetic reprogramming. We exposed F0 dams to HDM or saline during gestation and used the F1 males to generate F2 and F3 male progeny. A subset of each generation (F1, F2, and F3) were further challenged by HDM or saline, and subjected for the measurement of lung function, lung inflammation, and gene transcription/regulation (DNA methylation, DNA hydroxymethylation and mRNA expression). Here, we show that early life HDM exposure is capable of inducing multigenerational inheritance of lung phenotypes characterized by increased airway hyperresponsiveness, inflammation, and persistent changes in DNA methylation and gene expression up to at least the F3 generation.

### Animal Husbandry

All of the experimental procedures used were approved by the Institutional Animal Care and Use Committee of Johns Hopkins University (Baltimore, MD, United States) (MO17H187). Male and female C57Bl/6J mice were purchased from Jackson Laboratories (Bar Harbor, ME, United States). Mice were allowed to acclimate to housing at JHU for 2 weeks prior to the commencement of the experiment. All mice were maintained at 22°C, and 12-hour light and 12-hour dark cycle. The mice were housed in polysulfone-ventilated cages (Technoplast, Exton, PA, United States), and provided with Harlan Teklad Global 18% Protein Extruded Rodent Diet 2018SX and drinking water *ad libitum*.

## Materials and Methods

House dust mite extract (*D. Pteronyssinus*) was purchased from Greer (Lenoir, NC) in a lyophilized form. The level of HDM endotoxin was reduced using the EndoTrap HD assay (Hyglos GmbH, Germany) according to the protocol. Endotoxin level was measured using Pierce LAL Chromogenic Endotoxin Quantitation Kit (Thermo Scientific, Rockford, IL, United States). HDM extract was then diluted with phosphate buffered saline (PBS) to 2 mg/mL of total protein as measured by the Pierce BCA Protein Assay (Thermo Scientific Rockford, IL, United States).

### Early Life and Adult Exposures to HDM

This model of experimental allergic airway disease has been previously validated in our and W. Mitzner’s lab (Shang et al., 2013; Cheng et al., 2014; Wagner et al., 2015). Female nulliparous mice (F0) were sensitized to HDM (100 μg of protein) or an equivalent volume of PBS via intraperitoneal injection (i.p.) 2 weeks prior to timed mating, and exposed to HDM (100 μg of protein) or an equivalent volume of PBS via intratracheal instillation (i.t.) three times a week during pregnancy and lactation, starting at embryonic day E0.5 and continuing for 3 weeks post parturition. Instillation was assisted by the use of an isoflurane vaporizer and an induction chamber. Offspring were sex separated at 4 weeks of age and allowed to mature under the same housing conditions as mentioned in the Animal Husbandry section. Once the F1 male offspring reached 6 weeks of age, half of them were sensitized to HDM or saline (i.p) at day (d)0 and challenged with HDM or saline (i.t.) on d14, 18, and 21 before AHR measurement at d23.

### Generation of Sub-Generations

**FIGURE 1:**
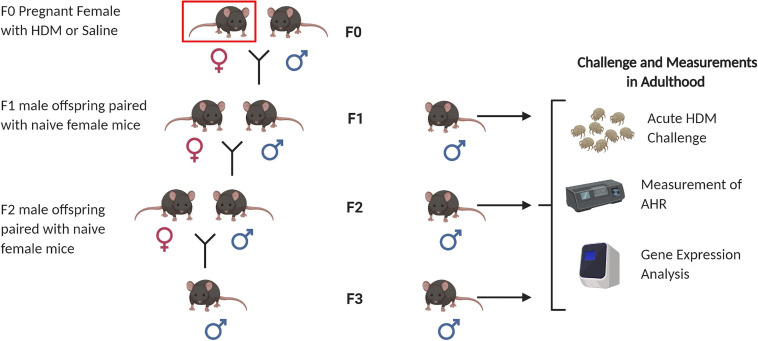
Animal breeding scheme for generation of F1, F2, and F3 progenies. Nulliparous C57BL/6J female mice (F0) were sensitized to house dust mite (indicated by red box) at 8 weeks of age, and then paired with naïve C57BL/6J male mice to generate the F1 progeny. F1 males were paired with naïve C57BL/6J female mice to generate F2 progeny. F2 males were paired with naïve C57BL/6J female mice to generate F3 progeny. A subset of 6 week-males from each generation (F1, F2, and F3) were selected for adult HDM challenge followed by airway hyperresponsiveness (AHR) measurements, and gene expression analysis. Figure created with BioRender.com.

Male F1 mice were paired with female naïve C57Bl/6J mice at 6 weeks to produce the F2 progeny. F3 were bred in the same manner. We summarized the breeding scheme in Figure 1. The male progenies from F2 and F3 were subject to acute HDM exposure when they reached 6-week old, via the same methods described for F1 male.

### Measurement of Airway Hyperresponsiveness (AHR)

Mice were administered ketamine/xylazine (25 mg/ml ketamine, 5 mg/ml xylazine) via intraperitoneal (i.p.) injection and once sedated and non-responsive to toe pinch they were placed on a heating pad. Mice were then intubated with a 20 g blunt cannula via tracheostomy. Lung phenotypic measurements, including airway resistance, were then assessed via flexiVent (SCIREQ, Montreal, Canada). Mice were maintained on the ventilator and paralyzed with succinylcholine (20 mg/ml) followed by methacholine challenge. Mice were exposed to increasing doses (0, 1, 3, 10, 30 mg/ml) of aerosolized methacholine chloride (Mch) (Sigma-Aldrich, St. Louis, MO, United States). The AHR was assessed by the change in pulmonary resistance (cmH2O.s/mL) as compared to the baseline using flexiVent (SCIREQ, Montreal, Canada) (Ewart et al., 1995).

### Collection of BALF and Tissue

After measurement on the flexiVent, mice were removed from ventilation, and quickly exsanguinated. Bronchoalveolar lavage (BAL) fluid was then collected by flushing the lungs with 1 mL of cold PBS with complete, Mini Protease Inhibitor Cocktail (Roche, San Luis Obispo, CA, United States). BAL was processed the same day, total cell count was conducted and slides were prepared using Cytospin. Organs were then dissected and flash frozen in liquid nitrogen and stored at −80°C until analysis.

#### Hydroxymethylated and Methylated DNA Immunoprecipitation Coupled With Next Generation Sequencing (hMeDIP- and MeDIP- seq)

DNA was isolated from lung tissues using AllPrep DNA/RNA/miRNA Universal Kit (QIAGEN, Germantown, MD, United States), according to manufacturer’s instructions. Sample DNA concentrations were quantified with the Quant–iT^TM^ Picogreen dsDNA assay kit (Invitrogen, CA, United States). A second quality check was done on Agilent 2100 HS DNA chip after DNAs were fragmented. 1 μg of DNA from each sample was fragmented on Covaris S2 and had an average length of 200 bp. Hydroxymethylated and methylated DNA was captured using the hMethylCap and MethylCap kit (Diagenode, NJ, United States) respectively. DNA was eluted from the protein complex. A total of 250 ng fragmented DNAs were amplified, ligated with linkers, and subjected to flow cells using the HiSeq2000 platform according to the manufacturer’s NGS protocol (Illumina, CA, United States). About 20 million reads obtained by a single-run of massive parallel sequencing with 51 bp paired-end reads were found. Bioinformatics analysis on the raw sequencing data was performed with use of CLC Genomic Workbench 6.0.3 (CLC Bios, MA, United States) and followed manufacturer’s standard data import protocol. Reads (peaks) were mapped to the Mouse Reference Genome Build NCBI38/mm10. Significant peaks by threshold profiles at a height equivalent to an estimated false discovery rate (FDR) 0.001 were identified. Differentially methylated regions and differential hydroxymethylated regions between HDM-exposed and saline-exposed mice were annotated to their chromosomal loci and Gene Ontology (GO) categories. The next generation sequencing (NGS) data generated in this project is deposited to the public (GEO accession number GSE169355).

### Real-Time Reverse-Transcriptase-PCR (RTPCR)

Total RNA (1 μg) was isolated from lung tissues using AllPrep DNA/RNA/miRNA Universal Kit (QIAGEN, Germantown, MD, United States), according to manufacturer’s instructions and reverse transcribed with iScript Reverse Transcriptase (BIO-RAD, Hercules, CA, United States). mRNA levels of the genes were quantified by TaqMan-based or SYBR Green-based real-time PCR. Primers were listed in Table 7. The 2^–ΔΔ*Ct*^ method was used to calculate the relative expression level of transcripts normalized to *Rpl19* (Shang et al., 2013).

### Statistical Analysis

Data on airway responsiveness, cell counts and relative gene expression were expressed as the mean- ± standard error of mean (SEM) with six mice per treatment group. Technical triplicates were performed in each assay. *P* values derived from Ordinary One-Way ANOVA with Tukey’s test to correct for multiple comparisons. ^∗^*P* < 0.05, ^∗∗^*P* < 0.01, ^∗∗∗^*P* < 0.001, ^****^P < 0.0001 in comparison with control indicates results were statistically significant. All data were analyzed and plotted with Prism8 (GraphPad Prism version 8.4.3 for Windows, GraphPad Software, La Jolla, CA, United States)^1^.

## Results

### Effect of Maternal HDM Exposure on Lung Pathophysiology of the F1 Progenies

**FIGURE 2:**
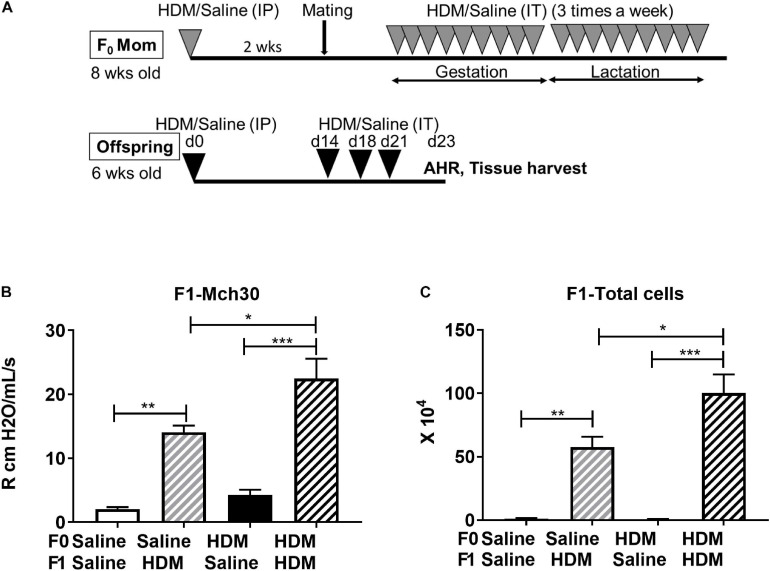
Effect of maternal exposure to HDM on sub-generation. **(A)** Experimental protocol for acute HDM exposure-induced AHR in a mouse model. Nulliparous C57BL/6J female mice (F0), 8-week old, were sensitized with 100 μg HDM or saline (as control) intraperitoneally (i.p.) 2 weeks before conception. Subsequently, these mice were mated with naive C57BL/6J males and then challenged with 100 μg HDM or saline intratracheally (i.t.) three times a week throughout gestation and lactation (total of 6 weeks). F1 offspring were sex separated at weaning. A subset of F1 males were unexposed and mated to naïve, nulliparous C57BL/6J female mice to produce F2 progeny, additionally, a subset of F2 males were left unexposed and mated to naïve, nulliparous C57BL/6J female mice used to produce F3 progeny. Additionally, a subset of F1, F2, and F3 males were subject to acute HDM exposures to examine multigenerational effects of HDM in mice. At 6 weeks, these offspring were sensitized with 100 μg HDM or saline (as control) intraperitoneally (i.p.) on Day (d)0. On d14, mice were challenged with 100 μg HDM or saline intratracheally (i.t.) 3× a week (d14, d18, and d21). On d 23, after airway responsiveness in response to methacholine (Mch) was assessed, lung tissue, blood serum, and bronchoalveolar lavage (BAL) fluid were collected. **(B)** Mice were administered methacholine (Mch), a bronchoconstrictive agent, at increasing doses (0.1, 0.3, 1, 3, 10, and 30 mg/ml) by a 10-second aerosol inhalation. Resistance at 30 mg/ml of Mch is shown. HDM-challenged mice exhibited increased AHR compared to those exposed to PBS. This was further enhanced by maternal HDM exposure. **(C)** Total cell count was performed after staining of BAL cells with Turks solution. HDM-challenged mice exhibited increased BAL cell number compared to those exposed to saline. This was further enhanced by maternal HDM exposure. Mean with SEM shown. *P* values derived from Ordinary One-Way ANOVA with Tukey’s test for multiple comparisons. *P < 0.05, **P < 0.01, ***P < 0.001, ****P < 0.0001.

F1 progenies were examined for allergy induced AHR when they reached 6-week old (Figure 2A). In response to adult HDM challenges, F1 progenies from dams exposed to saline or HDM (F0-Saline_F1-HDM or F0-HDM_F1-HDM) had significantly increased (*P* < 0.01) airway resistance during methacholine challenge (Figure 2B), as well as significantly increased cellularity in the bronchoalveolar lavage fluid (BALF), when compared to their counterparts who did not received HDM challenges in their adulthood (F0-Saline_F1-Saline or F0-HDM_F1-Saline) (Figure 2C). These changes were accompanied by increased expression or a trend in induction of cyclin D1 (*Ccnd1)*, proliferating cell nuclear antigen (*Pcna*) and calcium/calmodulin dependent protein kinase II delta (*Camk2dI*) (Table 1). These changes suggest that the adult acute exposure to HDM alters lung cell functions (including cell growth, cell proliferation and cell contraction) which may contribute to increased airway reactivity and airway inflammation. When we assessed the effect of maternal only HDM exposure on F1 progenies (F0-Saline_F1-Saline vs. F0-HDM_F1-Saline), we did not observe a statistically significant difference in airway reactivity, airway inflammation, or expression of AHR phenotypic genes. In the absence of maternal HDM exposure, F1 progenies (F0-Saline_F1-HDM) did not show increased collagen synthesis; collagen, type I, alpha 1 chain and Collagen, type III, alpha 1 chain (*Col1a1* and *Col3a*); in response to the adult HDM challenges. However, F1 progenies administrated to maternal HDM exposure (F0-HDM_F1-HDM) displayed significant upregulation of *Col1a* and a trend of induction of *Col3a* upon the exposure to HDM in their adulthood. In addition, there was a further HDM-induction of *Col3a* by 80% as compared to those from saline-exposed mom (vs. F0-Saline_F1-HDM). This change was associated with the further induction of AHR and production of immune cells by 70%. These results indicate maternal exposure to HDM alone did not alter the lung function of the offspring, but may promote the AHR in offspring who received the HDM challenge in their later life.

**TABLE 1:** mRNA levels of AHR phenotypic genes in F1 progenies.

	**A**		**B**			**C**			**D**			
	**F0-Saline_F1 Saline**		**F0-Saline_F1 HDM**			**F0-HDM_F1-Saline**			**F0-HDM_ F1 = HDM**			
**Gene**	**Mean**	**SEM**	**Mean**	**SEM**	***p* value (B vs. A)**	**Mean**	**SEM**	***p* value (C vs. A)**	**Mean**	**SEM**	***p* value (D vs. C)**	***p* value (D vs. B)**
Ccnd1	1.838	0.708	26.169	4.708	**0.039**	1.339	0.206	0.899	28.630	4.786	0.072	0.981
Pcna	0.353	0.040	4.483	0.949	0.065	1.896	0.592	0.279	5.225	0.509	**0.045**	0.897
Col1a	0.285	0.092	0.486	0.121	0.587	0.346	0.036	0.919	0.666	0.027	**0.009**	0.545
Col3a	0.638	0.178	1.087	0.127	0.310	1.071	0.251	0.563	2.003	0.187	0.136	0.056
Muc5b	1.168	0.118	1.053	0.108	0.885	1.156	0.120	1.000	1.276	0.175	0.937	0.719
Sma	0.347	0.043	0.443	0.103	0.824	0.422	0.166	0.967	0.806	0.114	0.363	0.212
Camk2d	3.537	0.309	7.137	0.544	**0.011**	3.387	0.137	0.966	8.666	0.979	0.077	0.587
Mylk	1.146	0.187	1.230	0.043	0.967	0.989	0.160	0.914	1.164	0.094	0.787	0.913

*mRNA levels measured via RTPCR. The 2∧(−ΔΔC_*t*_) method was used to calculate the relative expression level of transcripts normalized to Rpl19. *P* values derived from Ordinary One-Way ANOVA with Tukey’s test for multiple comparisons. Significant *P* values are in bold.*

### Maternal Exposures to HDM Reprogram F1 Progenies’ Lung Function, Through Epigenetic Modifications of Gene Transcription

We sought to test the hypothesis that maternal exposures to HDM increase offspring susceptibility to AHR by modulating lung gene expression via DNA methylation, given the fact that early-life epigenetic changes may modify lung cell function and later-life response. We previously reported that acute HDM challenges increased global 5-methylcytosine (5mC) and 5-hydroxymethylcytosine (5hmC) levels in mouse lung tissues (Shang et al., 2013). Herein, we applied MeDIP-seq and hMeDIP-seq to identify differential DNA methylation and hydroxymethylation changes in F1 progenies. 107 candidates and 118 candidates were shown differentially methylated and hydroxymethylated, respectively, when F1 progenies that received both maternal and adult exposure were compared with those that received adult HDM exposure only (F0-Saline_F1-HDM vs. F0-HDM_F1-HDM). Among these candidates, 26 genes were found in common (Table 2). Next, we examined if these differential epigenetic changes contribute to the changes in gene expression and increased AHR susceptibility in F1 progenies who received HDM exposures during gestation and lactation, and adulthood (Table 3 and Supplementary Table 1). Among these 26 genes, changes in mRNA level of erythroid differentiation regulator 1 (*Erdr1*, *p* < 0.001), potassium voltage-gated channel, subfamily H member 1 (*Kcnh1*, *p* < 0.001), non-protein coding RNA repressor of NFAT (*Nron*, *p* = 0.058) and sperm associated antigen 17 (*Spag17*, *p* < 0.001) in lung of HDM-exposed mice were concordant with the change in DNA methylation status, as well as the further induction of airway reactivity (F0-Saline_F1-HDM vs. F0-HDM_F1-HDM). Mice exposed to HDM during gestation and lactation showed a further increase in their gene expression (*Kchn1*, *Nron*, and *Spag17)* when they were challenged by HDM in adulthood. In contrast, maternal exposure to HDM reversed the effect of adult HDM exposure on induction of *Erdr1*. Another three gene candidates (dpy-19 like C-mannosyltransferase 1, *Dpy19l1*; leucyl-tRNA synthetase, *Lars;* and 2′-5′ oligoadenylate synthetase-like 2, *Oasl2*) were overexpressed in progenies that received adult HDM challenge, regardless of their maternal exposures (F0-Saline_F1-HDM and F0-HDM_F1-HDM). However, there was no additional induction of gene expression in mice exposed to HDM both *in utero* and in adulthood. On the other hand, we did not observe any significant change in mRNA levels of these 26 gene candidates in mice only exposed to HDM during gestation and lactation. Results suggest that the adult exposure to HDM may induce epigenetic alterations and/or advance the epigenetic effect of early-life HDM exposures on gene-specific expression, and its associated lung phenotypes in later life.

**TABLE 2:** List of differential hydroxymethylated and methylated genes associated with maternal HDM exposures and increased AHR.

**Gene Symbol**	**Gene Name**	**GO function**	**Chromosome**
Acox2	Acyl-Coenzyme A oxidase 2, branched chain	Signaling receptor binding and oxidoreductase activity, acting on the CH-CH group of donors	14
Aida	Axin interactor, dorsalization associated	protein domain specific binding	1
Asmt	Acetylserotonin O-methyltransferase	Protein homodimerization activity and methyltransferase activity	X
Cdr1	Cerebellar degeneration related antigen 1	Protein binding	X
Col23a1	Collagen, type XXIII, alpha 1	Extracellular matrix structural constituent, protein binding	11
Dpcd	Deleted in primary ciliary dyskinesia	Protein binding	19
Dpy19l1	dpy-19-like 1	Transferase activity, transferring glycosyl groups and mannosyltransferase activity	9
Eef2	Eukaryotic translation elongation factor 2	Protein kinase binding	10
Erdr1	Erythroid differentiation regulator 1	Not available	Y
Foxi1	Forkhead box I1	DNA-binding transcription factor activity, RNA polymerase II proximal promoter sequence-specific DNA binding	11
Kcnh1	Potassium voltage-gated channel, subfamily H (eag-related), member 1	Signal transducer activity and ion channel activity	1
Kdm6a	Lysine (K)-specific demethylase 6A	Protein binding and dioxygenase activity	X
Lars2	Leucyl-tRNA synthetase, mitochondrial	Binding and aminoacyl-tRNA editing activity	9
Luzp2	Leucine zipper protein 2	Extracellular region	7
Mrs2	MRS2 magnesium transporter	Magnesium ion transmembrane transporter activity	13
Muc19	Mucin 19	Extracellular region, Golgi lumen, plasma membrane	15
Nox3	NADPH oxidase 3	Oxidoreductase activity and superoxide-generating NADPH oxidase activity	17
Nron	Non-protein coding RNA, repressor of NFAT	Not available	2
Oasl2	2′-5′ oligoadenylate synthetase-like 2	RNA binding and transferase activity	5
Ranbp3l	RAN binding protein 3-like	Contributes to GTPase activator activity, Ran GTPase binding, SMAD binding	15
Rnu6	U6 small nuclear RNA	Not available	17
Smpdl3a	Sphingomyelin phosphodiesterase, acid-like 3A	Hydrolase activity and sphingomyelin phosphodiesterase activity	10
Spag17	Sperm associated antigen 17	Extracellular region, cytoplasm, cytoskeleton, microtubule, cilium	3
Tmem125	Transmembrane protein 125	Membrane, integral component of membrane	4
Ugt8a	UDP galactosyltransferase 8A	Carbohydrate binding and glucuronosyltransferase activity	3
Xcr1	Chemokine (C motif) receptor 1	G protein-coupled receptor activity and chemokine receptor activity	9

*Hydroxymethylated and methylated DNA was captured using the hMethylCap and MethylCap kit. Significant peaks by threshold profiles at a height equivalent to an estimated false discovery rate (FDR) 0.001 were identified. Differentially methylated and hydroxymethylated regions between HDM-exposed and saline-exposed mice were annotated to their chromosomal loci and Gene Ontology (GO) categories.*

**TABLE 3:** mRNA levels of differential hydroxymethylated and methylated genes in F1 progenies. mRNA levels measured via RTPCR.

	**A**		**B**			**C**			**D**			
	**F0 Saline F1 Saline**		**F0 Saline F1 HDM**			**F0 HDM F1 Saline**			**F0 HDM F1 HDM**			
**Gene**	**Mean**	**SEM**	**Mean**	**SEM**	***p* value (B vs. A)**	**Mean**	**SEM**	***p* value (C vs. A)**	**Mean**	**SEM**	***p* value (D vs. C)**	***p* value (D vs. B)**
Dpy19l1	1.125	0.387	57.229	8.803	**<0.001**	1.205	0.339	>0.9999	41.083	8.178	**<0.001**	0.299
Erdr1	1.235	0.282	57.112	4.660	**<0.001**	0.282	0.054	1.000	15.180	2.583	0.432	**<0.001**
Kcnh1	0.938	0.087	263.672	24.479	**<0.001**	4.918	1.222	0.978	501.429	7.045	**<0.001**	**<0.001**
Lars2	0.175	0.043	32.978	7.785	**0.003**	0.435	0.033	>0.9999	34.603	7.404	**0.004**	0.998
Nron	0.455	0.388	24.942	3.301	**0.042**	0.297	0.143	>0.9999	48.291	3.409	**<0.001**	0.058
Oasl2	0.461	0.052	33.065	3.284	**0.003**	0.459	0.015	>0.9999	50.182	3.798	**<0.001**	0.249
Spag17	0.633	0.007	182.749	27.169	**<0.001**	2.478	0.345	0.998	323.945	42.425	**<0.001**	**<0.001**

*The 2∧(−ΔΔC_*t*_) method was used to calculate the relative expression level of transcripts normalized to Rpl19. *P* values derived from Ordinary One-Way ANOVA with Tukey’s test for multiple comparisons. Significant *P* values are in bold.*

To examine if these gene-specific epigenetic changes were accompanied with changes in epigenetic modification enzymes in response to HDM (Cheng et al., 2014), we measured the expression of these enzymes (Table 4). F1 progenies who solely received exposure to HDM in adulthood (F0-Saline_F1-HDM and F0-HDM_F1-HDM) showed a reduction in DNA methyltransferase 3b (*Dnmt3b*) and methyl CpG binding protein 2 (*Mecp2*), when compared to their counterparts not exposed to HDM in adulthood. This change was not found in mice only exposed to HDM during gestation and lactation. There was no additional reduction in *Dnmt3b* and *Mecp2* expression in F1 progenies who received both maternal and adult challenges to HDM. F1 progenies who solely received exposure to HDM in gestation and lactation (F0-HDM_F1-Saline) showed decreased expression of DNA methyltransferase 1 (*Dnmt1*) and DNA methyltransferase 3a (*Dnmt3a*) by 2.4 and 14-fold, respectively, when compared to those from saline-exposed dams. However, F1 progenies that received both maternal HDM exposure and adult HDM exposure did not show further changes in expression of these enzymes, which function in DNA methylation. Next, we examined the effect of HDM on the expression of enzymes facilitating DNA hydroxymethylation; tet methylcytosine dioxygenase (Tet) 1 (*Tet1*) and *Tet2*. A 3-fold overexpression of *Tet1*, but no change in *Tet2*, was found in F1 progenies that received adult HDM challenges. Results indicate that the overexpression of identified gene candidates (Table 3) may correlate to the changes in these epigenetic modification enzymes, although further study is required to demonstrate how these epigenetic modification enzymes alter the gene transcription.

**TABLE 4:** mRNA levels of epigenetic modification enzymes in F1 progenies. mRNA levels measured via RTPCR.

	**A**		**B**			**C**			**D**			
	**F0 Saline F1 Saline**		**F0 Saline F1 HDM**			**F0 HDM F1 Saline**			**F0 HDM F1 HDM**			
**Gene**	**Mean**	**SEM**	**Mean**	**SEM**	***p* value (B vs. A)**	**Mean**	**SEM**	***p* value (C vs. A)**	**Mean**	**SEM**	***p* value (D vs. C)**	***p* value (D vs. B)**
Dnmt1	1.518	0.595	0.013	0.000	**0.003**	0.445	0.117	0.073	0.004	0.002	0.736	>0.9999
Dnmt3a	3.671	0.503	0.182	0.023	**<0.001**	0.234	0.061	**<0.001**	0.002	0.001	0.949	0.970
Dnmt3b	2.975	0.381	0.108	0.032	**<0.001**	2.019	0.389	0.131	0.014	0.005	**<0.001**	0.996
Mecp2	2.412	0.248	0.906	0.163	**0.002**	1.518	0.066	0.174	0.119	0.003	**0.010**	0.219
Tet1	0.668	0.029	3.910	0.647	**<0.001**	0.825	0.165	0.983	4.450	0.184	**<0.001**	0.542
Tet2	1.233	0.358	1.504	0.341	0.907	1.165	0.262	0.999	1.081	0.361	0.997	0.720

*The 2∧(−ΔΔC_*t*_) method was used to calculate the relative expression level of transcripts normalized to Rpl19. *P* values derived from Ordinary One-Way ANOVA with Tukey’s test for multiple comparisons. Significant *P* values are in bold.*

### Multigenerational Effect of F0 HDM Exposure on Lung Pathophysiology of the F2 and F3 Progenies

**FIGURE 3:**
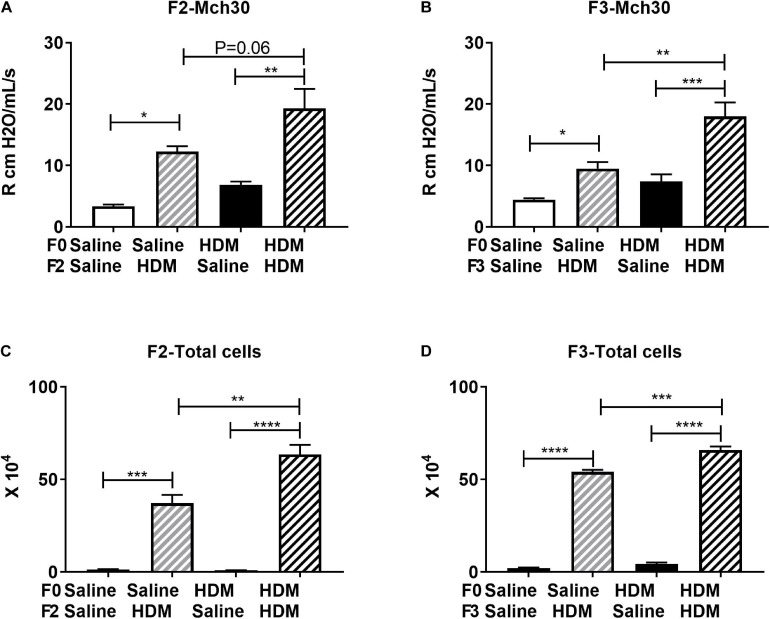
F0 HDM exposure enhances severity to HDM induced airway hyperresponsiveness and cell count in BAL in F2 and F3. **(A)** F2 Resistance at 30 mg/ml of Mch is shown. F2 progeny from HDM-challenged mice exhibited increased AHR compared to those exposed to PBS. This was further enhanced by maternal HDM exposure. **(B)** F3 Resistance at 30 mg/ml of Mch is shown. F3 progeny from HDM-challenged mice exhibited increased AHR compared to those exposed to PBS. This was further enhanced by maternal HDM exposure. **(C)** F2 progeny from HDM-challenged mice exhibited increased BAL cell number compared to those exposed to saline. This was further enhanced by maternal HDM exposure. **(D)** F2 progeny from HDM-challenged mice exhibited increased BAL cell number compared to those exposed to PBS. This was further enhanced by maternal HDM exposure. Mean with SEM shown. *P* values derived from Ordinary One-Way ANOVA with multiple comparisons. *P < 0.05, **P < 0.01, ***P < 0.001, ****P < 0.0001.

We next examined the same parameters of allergic-induced airway reactivity and inflammation measured in the F1 progenies, in the F2 and F3 progenies. We found that the effect of maternal exposure to HDM on inheritance of the allergic responses carried over. In response to adult exposure to HDM, F2, and F3 progenies from either saline- or HDM-exposed F0 (F0-Saline_F2/3-HDM and F0-HDM_F2/3-HDM) showed increased airway reactivity (increased lung resistance in response to methacholine) and airway inflammation (induction of total number of immune cells) (Figure 3). These changes were accompanied by a significant induction of *Ccnd1*, *Pcna*, *Col3a*, and *Camk2d* (Table 5). These findings were quite similar to those observed in the in the F1 progenies. Like in the F1, without the challenge of HDM in the adulthood, there was no significant change in airway reactivity and airway inflammation in F2 and F3 progenies from grand-maternal (F0) exposure to HDM (F0-HDM_F2-Saline and F0-HDM_F3-Saline) (Figure 3) although these progenies showed increased expression of *Col1a* and *Camk2d*, when compared to their counterparts from saline-exposed dams (F0) (Table 5). With the addition of acute HDM exposure during adulthood, F2 and F3 progenies from grand-maternal (F0) exposure to HDM (F0-HDM_F2-HDM and F0-HDM_F3-HDM) showed a further induction of allergic response to methacholine challenge and airway inflammation, when compared to those from saline-exposed dams (F0) (Figure 3). This change was accompanied by an increased expression of *Col3a* in F2 progenies (*p* = 0.006) and a trend of induction of *Col3a* in F3 progenies (Table 5). The increased expression of *Col3a* potentially indicates the increased airway reactivity and inflammation via increased collagen expression. Taken together, these results suggest the multigenerational effect of the early-life exposure to HDM on offspring asthma risk, through alteration of the lung phenotypic responses.

**TABLE 5:** mRNA levels of AHR phenotypic genes in F2 and F3 progenies. mRNA levels measured via RTPCR.

**F2 Generation**												
	**A**		**B**			**C**			**D**			
	**F0 Saline F1 Saline**		**F0 Saline F1 HDM**			**F0 HDM F1 Saline**			**F0 HDM F1 HDM**			
**Gene**	**Mean**	**SEM**	**Mean**	**SEM**	***p* value (B vs. A)**	**Mean**	**SEM**	***p* value (C vs. A)**	**Mean**	**SEM**	***p* value (D vs. C)**	***p* value (D vs. B)**
Ccnd1	1.679	0.509	14.755	2.471	0.076	1.069	0.118	0.692	17.372	5.062	0.200	0.962
Pcna	0.219	0.013	4.630	1.245	0.171	0.485	0.234	0.710	3.132	0.212	**0.004**	0.686
Col1a	0.749	0.121	1.336	0.204	0.230	0.138	0.008	0.090	1.427	0.080	**0.009**	0.972
Col3a	0.416	0.120	1.478	0.114	**0.011**	0.676	0.092	0.427	3.143	0.163	**0.002**	**0.006**
Muc5b	1.304	0.322	1.030	0.055	0.836	1.330	0.117	1.000	1.247	0.345	0.995	0.917
Sma	1.031	0.328	1.486	0.323	0.765	1.175	0.182	0.978	1.645	0.169	0.357	0.968
Camk2d	3.424	0.059	5.152	0.421	0.128	2.208	0.091	**0.003**	5.381	0.737	0.118	0.992
Mylk	1.332	0.081	1.343	0.051	1.000	0.974	0.130	0.253	1.074	0.149	0.953	0.472

**F3 Generation**												

	**A**		**B**			**C**			**D**			
	**F0 Saline F1 Saline**		**F0 Saline F1 HDM**			**F0 HDM F1 Saline**			**F0 HDM F1 HDM**			

**Gene**	**Mean**	**SEM**	**Mean**	**SEM**	***p* value (B vs. A)**	**Mean**	**SEM**	***p* value (C vs. A)**	**Mean**	**SEM**	***p* value (D vs. C)**	***p* value (D vs. B)**
Ccnd1	5.201	0.937	32.478	7.568	0.072	3.886	0.880	0.742	29.012	3.948	**0.020**	0.975
Pcna	0.682	0.231	4.419	0.338	**<0.001**	0.871	0.135	0.892	4.092	0.646	**0.041**	0.967
Col1a	1.958	0.305	3.952	0.909	0.278	3.254	0.183	**0.038**	3.303	0.561	1.000	0.926
Col3a	0.383	0.103	1.630	0.229	**0.012**	0.367	0.082	0.999	1.948	0.085	**<0.001**	0.599
Muc5b	2.788	0.307	4.797	0.825	0.220	3.079	0.422	0.941	4.572	0.692	0.356	0.997
Sma	1.116	0.165	2.130	0.333	0.121	0.974	0.134	0.907	1.905	0.265	0.099	0.949
Camk2d	3.379	0.592	6.465	0.792	0.061	2.232	0.140	0.349	3.979	0.417	0.061	0.114
Mylk	1.410	0.091	3.461	0.586	0.078	1.509	0.080	0.844	2.934	0.308	**0.049**	0.855

*The 2∧(−ΔΔC_*t*_) method was used to calculate the relative expression level of transcripts normalized to Rpl19. *P* values derived from Ordinary One-Way ANOVA with Tukey’s test for multiple comparisons. Significant *P* values are in bold.*

### Multigenerational Inheritance of Epigenetic Reprograming in F2 and F3 Progenies

The inheritance of allergic-induced lung phenotypic responses in the F2 and F3, suggests that the airway reactivity and the increased cellularity phenotype, as well as the expression of markers of AHR are capable of multigenerational transmission. We examined if this could be explained by the epigenetic reprogramming in F2 and F3 progenies from grandmaternal (F0) HDM exposure by assessing any differential expression of genes identified by NGS (Table 6 and Supplementary Tables 1, 2). We showed that F1 progenies from dams (F0) exposed to HDM showed a further induction in expression of *Kcnh1*, *Nron* and *Spag17*. In the F2 progenies, the same induction of *Kcnh1* and *Spag17* (but not *Nron*) was seen (Table 6). *Kcnh1* was the only candidate gene that showed further induction in gene expression in the F3 progenies from grandmaternal (F0) HDM exposure. Our results suggest that the inheritance of the allergic phenotype could be induced by the epigenetic reprogramming of gene-specific transcription. However, these changes were not observed in F2 and F3 progenies who were not challenged by HDM in adulthood. It indicates early-life exposure to HDM did not induce a dramatic increase in allergic responses seen in the sub-generations, but may induce epigenetic programing, which may prime the offspring’s lung genome to be more susceptible upon a second-hit of the exposure, leading to the development of a pronounced increased allergic responses and AHR.

**TABLE 6:** mRNA levels of differential hydroxymethylated and methylated genes in F2 and F3 progenies.

**F2**												
	**A**		**B**			**C**			**D**			
	**F0 Saline F1 Saline**		**F0 Saline F1 HDM**			**F0 HDM F1 Saline**			**F0 HDM F1 HDM**			
**Gene**	**Mean**	**SEM**	**Mean**	**SEM**	***p* value (B vs. A)**	**Mean**	**SEM**	***p* value (C vs. A)**	**Mean**	**SEM**	***p* value (D vs. C)**	***p* value (D vs. B)**
Kcnh1	3.516	0.833	257.744	49.664	**<0.001**	3.588	0.133	>0.9999	374.755	112.999	**<0.001**	**<0.001**
Spag17	1.533	0.202	170.063	42.397	**<0.001**	1.457	0.317	>0.9999	121.977	3.923	**<0.001**	0.058

**F3**												

	**A**		**B**			**C**			**D**			
	**F0 Saline F1 Saline**		**F0 Saline F1 HDM**			**F0 HDM F1 Saline**			**F0 HDM F1 HDM**			
**Gene**	**Mean**	**SEM**	**Mean**	**SEM**	***p* value (B vs. A)**	**Mean**	**SEM**	***p* value (C vs. A)**	**Mean**	**SEM**	***p* value (D vs. C)**	***p* value (D vs. B)**
Kcnh1	5.106	0.661	366.325	51.904	**<0.001**	2.137	0.598	0.997	471.418	77.886	**<0.001**	**<0.001**
Spag17	3.589	0.370	138.982	32.860	**<0.001**	1.844	0.182	0.999	160.806	26.801	**<0.001**	0.464

*mRNA levels measured via RTPCR. The 2∧(−ΔΔC_*t*_) method was used to calculate the relative expression level of transcripts normalized to Rpl19. *P* values derived from Ordinary One-Way ANOVA with Tukey’s test for multiple comparisons. Significant *P* values are in bold.*

**TABLE 7:** Sequence of primers for qPCR.

**Gene Name**	**Primer Sequence-Forward**	**Primer Sequence-Reverse**
Acox2	TCCAGAAGGCTTGCACCATT	TTTGCCTCTGGGTCACTAGG
Aida	CGCCGACTTCGACTCTTGG	TTTGCCTATGGTTTTCTTTTGTTCT
Asmt	CGCCATCTACAGGTCGGAG	GGTCGCAGATGACCCTGAAG
Camk2d	TaqMan Probe # mm0049926	
Ccnd1	ACCTGGGCAGCCCCAACAAC	GGAGGCAGTCCGGGTCACACT
Col1a	TTGGGTCCCTCGACTCCTAC	TGACTGTCCCACGTAAGCAC
Col23a	ACGGGAGAAGTTGGATGGAG	ATCTCGTCCTGATTGCCCTG
Col3a	GAGGGCCATAGCTGAACTGA	TGCAGAGTTAACAACAGTCAGC
Dpcd	CGAGCTCATCAAAGAAACCCA	CATCCTTCGGGTAGGGGAGA
Dpy19l1	GCCAGCTGGTACCGGATTTA	CCCAATCCCTCACAGCTCTC
Eef2	GTGGGGAGACCGGTACTTTG	AGAAGGTGCGGGGAAGTTTT
Erdr1	TTAGCCGCAGCTATGGTTTCT	TTCCATTCACGCCCACAGAG
Foxi1	ATGAGGACGACCCAGGCAAAG	TTCCTGCGAAAGTTTCCGTTG
Kcnh1	CTGACCCCAAACTTATCCGCA	CTGATGCCCTCATCCACGTTC
Kdm6a	AAACGCACCCACTCTACCTC	CCTTTGTGAAGCCCCTGAGT
Lars2	GGGTTTGGACCCAGAAAAGGA	GAAGACCCTTCTCTGTAAGCTGTG
Luzp2	TCAGCACCAGACAGGACTAT	TCTCTGGATGTCTTTGTCAGC
Mlck	TaqMan Probe # mm00653039	
Mrs	ACGGCAAAAGTCTCTCTGAGTT	TCAAAGACATGTGGGTCGCT
Muc19	TTGATGACCCAAGCAACCCA	TTGCTTTGGGCAGTCCTGAA
Muc5b	GAAACTGGAGCTGGGCTCTG	ATGGAGTCACTATACACTCTCTGA
Nox3	ACGGAGGAGGTCGCATCATT	GCCTGCCATTCAGCATAGTG
Nron	CACGGGTGCTGGATGACATA	ATTTAAGGGAGAGCTGGCGG
Oasl2	GAGACCGGCCCATCATCCT	CTACAGTCGTGCAACAGACCT
Pcna	CTGGGACGTCAGCTCGGGCG	TTGGACATGCTGGTGAGGTTCACG
Ranbp31	GTAGGCAGGAGGTGCGATAC	ACTCCTCGAAAAGCATGCCA
Rpl19	GGTGACCTGGATGAGAAGGA	TTCAGCTTGTGGATGTGCTC
Sma	CTTCTCCAGGGAGGAAGAGG	ACTACTGCCGAGCGTGAGAT
Smpd13a	AGCTGTGGGGCAGTTTTGG	CACACACCTTGGTACGGTCA
Spag17	CACCAACTGCGAGGACAGTA	GTAGCACCTGGTATGACCCC
Tmem125	CCTGTGTGAGAGGTGAGGGTA	TGTCAGGGTTCAGAGGGTGT
Ugt8a	TACAGGCAAAAGGCATGGGG	CCTCTGCCGATAACTGGGAT
Xcr1	GAGTCAGATGCTCTCAGTATCCCT	GGACAATGGTAGAGATGGTGGAA

## Discussion

In the present study, we observed that HDM exposure in adulthood results in the previously observed allergic airway disease phenotype (Shang et al., 2013; Cheng et al., 2014), characterized by increased AHR and increased cell number in BALF. This was accompanied by the increased expression of genes governing cell proliferation (*Ccnd1*) and force-generating capacity (*Camk2d*). Unlike our previous study, we did not observe any significant change in mRNA level of *Muc5b*, *Sma*, and *Mylk* in progenies with adult exposure to HDM. We suggest the acute exposure in the present study may partially explain this, as expression of these genes was observed in a chronic HDM exposure model that was accompanied by significant airway remodeling. Strikingly, maternal and adult exposures to HDM resulted in a synergistic effect in F1 progenies (F0-HDM_ F1-HDM) when compared to adult only exposure (F0-Saline_F1-HDM), characterized by increased AHR, airway inflammation, and collagen synthesis (increased expression of *Col3a*). These changes were accompanied by changes in gene-specific methylation and hydroxymethylation as revealed by MeDIP-seq and hMeDIP-seq analysis.

To investigate the role of maternal exposure on the synergistic phenotype, we compared the progenies that received both maternal and adult exposure to HDM (F0-HDM_F1-HDM) to those who solely received adult exposure to HDM (F0-Saline_F1-HDM). We focused on the genes which showed changes in both methylation and hydroxymethylation at their promoters. As increased hydroxymethylation and decreased methylation at the promoter, and vice versa, is known to contribute to the regulation of gene transcription, we measured the mRNA levels of these candidates to validate if these epigenetic changes contribute to changes in gene expression. Maternal and adult exposure to HDM resulted in a synergistic effect on the induction of *Kcnh1*, *Nron*, and *Spag17*, when compared to that of adult only exposure (F0-HDM F1-HDM vs. F0-Saline F1-HDM). Conversely, the induction of *Erdr1* expression by adult HDM exposure was reduced in progenies with maternal (F0) exposure to HDM. Besides *Erdr1*, *Kcnh1*, *Nron*, and *Spag17*, we did observe significant changes in DNA methylation and hydroxymethylation of other 22 gene candidates that associated with the increased AHR. However, we did not find concordance between the induction of gene expression and methylation changes of these 22 genes. This could be explained by mechanisms other than DNA base modification, underlying the transcription of genes, although we observed acute exposure to HDM induced alteration in expression of *Dnmt1*, *Dnmt3a*, *Dnmt3b*, *Mecp2*, and *Tet1*. Nevertheless, the methylation marks induced by HDM exposure may perhaps be used as the indicator for exposure, but not the phenotypic changes (gene expression and lung functions) if these marks are validated in other cohorts exposed to HDM.

*Kcnh1*, in particular, is of interest, as it has been linked to asthma-related outcomes. Recently, a study investigating environmental tobacco smoke (ETS) and lung function identified an interaction between a SNP in *KCNH1* and reduced FEV1 after ETS exposure (de Jong et al., 2017). Additionally, a study investigating season of birth with allergic outcomes identified a CpG site upstream of *KCNH1* as the mediator between spring birth dates and high serum IgE levels (Lockett et al., 2016). *KCHN1* encodes for a potassium voltage gated channel subunit that is important for smooth muscle contraction, thus it is possible that the increased expression of *KCNH1* is indicative a change in smooth muscle contractile machinery in the airways. *SPAG17* has been linked to the immune and lung related outcomes, as it has been shown to be associated with asthma susceptibility (Torgerson et al., 2012). *Spag17* is crucial for the proper function of the axoneme which forms the core of motile cilia, which are found in respiratory tract. Deletions or mutations of *Spag17* lead to immotile cilia in the nose and trachea and result in reduced mucociliary clearance, eventually leading to respiratory distress (Teves et al., 2013). We found a large increase (100-fold) in the expression of *Spag17* due to HDM exposure, which may be linked to a structural change in the airways. *Nron* is a long non-coding RNA (lncRNA) that is capable of modulating T cells at the transcriptional and protein level, as well as forming complexes with Nuclear factor of activated T cells (NFAT), and preventing NFAT translocation to the nucleus after the reception of cytokines (Xie and Liu, 2015). Given the immunological underpinnings of allergic disease and the increase in cell number in BALF, the large increase in *Nron* expression may reflect the expansion of T cell populations in the lung in response to HDM. Finally, *Erdr1* is a pleiotropic cytokine involved in hemoglobin synthesis, that has been implicated in several other disease states. In the intestine, *Erdr1* has been shown to promote Wnt signaling, leading to epithelial growth and improved wound closure after injury to the mucosa (Abo et al., 2020). Intriguingly, *Erdr1* has been shown to be upregulated under a variety of cell stress conditions and has been shown to be negatively correlated with IL-18 signaling (Dörmer et al., 2004; Kim et al., 2016). Future work can investigate the epigenetic regulation of these genes as well as quantify and localize the expression of their products. In addition, we showed that maternal exposure to HDM induced a further induction of *Col3a* in progenies that received adult exposure to HDM. We reported that hydroxymethylation of *COL3A* promoter was associated with the increased gene expression in human ASM cells from asthmatics (Yeung et al., 2020). Taken together, maternal exposure to HDM induces gene-specific epigenetic changes in offspring in genes associated with immune response, asthma severity, and lung function. These changes may promote the observed lung phenotypic changes and result in increased susceptibility to allergic AHR.

In direct comparison to the adult only exposure, we were intrigued to see that maternal exposure to HDM (F0-HDM_F1-Saline) did not produce an overt phenotype in F1 offspring when compared to F1 (F0- Saline_F1-Saline). This was despite work in our lab and in others showing that maternal exposures to HDM, nicotine, and air pollution, and phthalates can alter the lung function in the F1 and beyond (Rehan et al., 2013; Gregory et al., 2017; Jahreis et al., 2018). Additionally, there was no change in the expression of the identified gene candidates, when compared to control (F0-HDM_F1-Saline vs. F0-Saline_F1-Saline). However, we did find that maternal HDM exposure led to a decrease in expression of *Dnmt3a*. Based on these observations alone, one might think that the maternal exposure, or perhaps its timing or duration, buffers the offspring from the deleterious effects of HDM. However, we show that maternal and adult exposure to HDM in F1 progenies (F0-HDM_F1-HDM), resulted in a synergistic effect, characterized by increased AHR and expression of *Col3a*, *Kcnh1*, *Nron*, and *Spag17*, when compared to adult only exposure. We speculated that the early-life exposure to HDM in F1 (throughout gestation and lactation) may have served as a time of early immunological sensitization. Upon exposure to HDM as an adult this may have produced the significantly enhanced response we observed. Alternatively, this early-life exposure could have altered any number of pathways, including those related to metabolism, lung function, or immune response, predisposing the F1 to enhanced disease upon adult challenge to HDM. This phenomenon is akin to the “two-hit model,” in which the primary exposure predisposes the offspring to a phenotype, but a second exposure or stressor is required to initiate the phenotype (Crews et al., 2012).

We next showed that maternal exposure to house allergen can leave an epigenetic mark on subsequent descendants, resulting in an asthma-like disease transmitted along the paternal side. This effect occurred despite the fact that allergen exposure was limited to the F0 (intratracheal) and/or the F1 (during gestation and lactation). The F2 generation was generated from the paternal lineage (F1 males not exposed as adults) and naïve female mice, the offspring were never exposed to HDM before the adult, acute exposure to HDM. To our surprise, F2 progenies exposed to HDM in adulthood (F0-Saline_F2-HDM), displayed a similar phenotype as their F1 parent, characterized by significantly increased airway reactivity and increased cellularity in BALF. Furthermore, these changes were accompanied by changes in gene expression in the lung, including upregulation of *Col3a*, potentially indicating structural alterations, as well as *Kcnh1* and *Spag17* both of which were upregulated in the F1. Thus, our data indicate that early-life exposure to HDM is sufficient to transmit an asthma-like phenotype to the subsequent generation via the paternal line, and that this phenotype is accompanied by overt changes in epigenetic alterations. Similar to the F1 progenies that received saline in adulthood (F0-HDM_F1-Saline), the F2 progenies challenged with saline (F0-HDM_F2-Saline) did not display a discernable change in airway mechanics and airway inflammation. These findings support our 2-hit hypothesis, in which the maternal-only HDM exposure was not sufficient to drive a phenotype, yet resulted in altered epigenetic changes in the lung, perhaps increasing susceptibility to HDM in the event of a second insult.

Next, to examine the durability of this asthma-like phenotype, we looked at the subsequent generation, the F3, bred from F2 males (the F1 founders of this lineage only experienced HDM exposure *in utero*/early life when administered to F0) and naïve females, similarly F2 founders of this line were never exposed as adults. Here, we observed the extension of the phenotype observed in the F1 and F2. F3 progenies from paternal lineage with early-life HDM exposure showed significantly increased airway reactivity and airway inflammation, after adult, acute exposure to HDM. Like the previous generation (F2), they also displayed increased expression of *Col3a*, potentially indicative of structural changes in the airway. Additionally, they displayed increased expression of *Kcnh1*, but not *Spag17*. These findings are particularly striking as they point to multigenerational mechanisms of exposure leading to a durable genotype and phenotype. The persistence of this effect and the observed changes in expression of epigenetic modification enzymes certainly point to the epigenetic mechanisms as a possible mediator of this multigenerational effect. Furthermore, much like the F1, and F2 before them, the F3 from paternal lineage with solely maternal HDM exposure (F0-HDM_F3-Saline) showed no discernable change in AHR phenotypes and epigenetic alterations. The lack of an overt phenotype from maternal only exposure, but the clear indication of a synergistic effect upon adult exposure may hint at a role of maternal HDM exposure in priming the offspring to respond to a second insult.

In summary, HDM exposure is capable of producing multigenerational inheritance of AHR phenotype, as well as a range of transcriptional and epigenetic changes, along the paternal line, in the presence of a second insult. The second insult, acute HDM challenge, was sufficient to drive allergic airway disease pathogenesis in the F2 and F3 progenies, and resulted in a more severe disease phenotype than in adult only exposure (F0 Saline). These findings are similar to previous research in which mice exposed to diesel exhaust particles at F0 have offspring that display greater susceptibly to ovalbumin induced allergic airway disease. This increased susceptibility continued into the F2 and F3 generations, with reduced susceptibly in F3. Additionally, this susceptibility was associated with DNA methylation changes in dendritic cells, with distinct methylation patterns identified in F1, F2, and F3 (Gregory et al., 2017). Similar to our model, a second insult, in this case ovalbumin, elicited greater effects in indirectly exposed offspring. Given the similarities observed between the two models, it will be worth examining if other models of sensitization and challenge, or the use of other allergens, like cockroach or feline allergen, can produce similar multigenerational effects. Additionally, the susceptibility of these progeny to other challenges, like particulate matter or lipopolysaccharide, is worth investigating. In this study, we focused on the effect of HDM on epigenetic regulation of lung methylome because we and others reported that HDM, which is commonly found in household, induces several features of human asthma when administered to mice. Examination of shared epigenetic or transcriptomic features obtained from different exposure models could provide insight into shared underlying mechanisms of multigenerational inheritance. Our findings just scratch the surface of this phenomenon, as our understanding of the mechanisms of transgenerational inheritance are in their infancy. This does, however, make a strong case for the contribution of maternal exposures to offspring disease outcomes, including allergic airway disease. Given that we observed transgenerational effects along the paternal line, examination of the sperm, particularly miRNAs and DNA methylation and hydroxymethylation profiles are warranted. Additionally, the contribution of the maternal line to the AHR phenotype needs to be further investigated. Looking forward, identification of cell- and tissue-specific epigenetic mechanisms (such as Dnmt-dependent DNA methylation and/or Tet1-dependent DNA hydroxymethylation) responsible for the increase in expression of *Col3a*, *Kcnh1*, *Nron*, and *Spag17*, should provide a deeper insight into the molecular underpinnings of this effect.

## Data Availability Statement

The datasets presented in this study can be found in online repositories. The names of the repository/repositories and accession number(s) can be found below: https://www.ncbi.nlm.nih.gov/, GSE 169355.

## Ethics Statement

The animal study was reviewed and approved by Institutional Animal Care and Use Committee of Johns Hopkins University.

## Author Contributions

RC, YS, WM, and W-yT designed the study. NL, TD, and YS performed the lung physiological study. YS and TD performed the gene expression and methylation study with advice of W-yT and JP analyzed and interpreted the results and prepared the manuscript. All authors contributed to the article and approved the submitted version.

## Conflict of Interest

The authors declare that the research was conducted in the absence of any commercial or financial relationships that could be construed as a potential conflict of interest.

## Footnotes

1 www.graphpad.com
