# Supplementary material for: Multigenerational Epigenetic Regulation of Allergic Diseases: Utilizing an Experimental Dust Mite-Induced Asthma Model
Source: Front Genet. 2021 Apr 1;12:624561. doi: 10.3389/fgene.2021.624561 (PMC8047068; doi:10.3389/fgene.2021.624561)
Supplement: Supplementary file 1 [file Data_Sheet_1.docx]

**Supplemental Table**

**Supplemental Table 1:** mRNA levels of differential hydroxymethylated and methylated genes in F1, F2 and F3 progenies. mRNA levels measured via RTPCR. The 2^(–delta delta C_t_) method was used to calculate the relative expression level of transcripts normalized to Rpl19. P values derived from Ordinary One-Way ANOVA with Tukey’s test for multiple comparisons.

| **Supplemental Table 1: mRNA levels of differential hydroxymethylated and methylated genes in F1-F3 progenies** | | | | | | | | | | | | |
| --- | --- | --- | --- | --- | --- | --- | --- | --- | --- | --- | --- | --- |
| **F1** | | | | | | | | | | | | |
|  | **A** | | **B** | | | **C** | | | **D** | | | |
|  | **F0 Saline F1 Saline** | | **F0 Saline F1 HDM** | | | **F0 HDM F1 Saline** | | | **F0 HDM F1 HDM** | | | |
| **Gene** | **Mean** | **SEM** | **Mean** | **SEM** | **p value (B vs A)** | **Mean** | **SEM** | **p value (C vs A)** | **Mean** | **SEM** | **p value (D vs C)** | **p value (D vs B)** |
|  |  |  |  |  |  |  |  |  |  |  |  |  |
| Acox2 | 0.030 | 0.016 | 0.000 | 0.000 | >0.9999 | 0.016 | 0.015 | >0.9999 | 0.000 | 0.000 | >0.9999 | >0.9999 |
| Asmt | 0.386 | 0.106 | 15.213 | 3.063 | 0.375 | 0.120 | 0.038 | >0.9999 | 8.116 | 0.686 | 0.849 | 0.868 |
| Col23a1 | 2.054 | 0.304 | 2.349 | 0.370 | >0.9999 | 5.567 | 0.918 | 0.984 | 4.917 | 1.260 | 1.000 | 0.992 |
| Dpcd | 1.091 | 0.133 | 0.014 | 0.005 | 0.999 | 0.900 | 0.222 | >0.9999 | 0.005 | 0.003 | 1.000 | >0.9999 |
| Eef2 | 0.850 | 0.103 | 0.098 | 0.023 | 1.000 | 0.833 | 0.097 | >0.9999 | 0.032 | 0.025 | 1.000 | >0.9999 |
| Kdm6a | 0.669 | 0.087 | 0.210 | 0.042 | >0.9999 | 0.472 | 0.027 | >0.9999 | 0.110 | 0.017 | >0.9999 | >0.9999 |
| Luzp2 | 0.986 | 0.099 | 0.068 | 0.013 | 1.000 | 1.055 | 0.124 | >0.9999 | 0.009 | 0.004 | 1.000 | >0.9999 |
| Mrs2 | 0.203 | 0.035 | 1.002 | 0.101 | 1.000 | 0.243 | 0.038 | >0.9999 | 1.434 | 0.142 | 0.999 | >0.9999 |
| Muc19 | 0.014 | 0.003 | 5.628 | 1.307 | 0.929 | 0.030 | 0.006 | >0.9999 | 10.825 | 1.533 | 0.692 | 0.943 |
| Nox3 | 0.735 | 0.084 | 20.338 | 3.629 | 0.148 | 0.772 | 0.036 | >0.9999 | 12.501 | 4.251 | 0.633 | 0.830 |
| Ranbp3l | 0.000 | 0.000 | 0.106 | 0.015 | >0.9999 | 0.091 | 0.044 | >0.9999 | 1.496 | 0.186 | 0.999 | 0.999 |
| Smpdl3a | 0.309 | 0.062 | 7.361 | 0.499 | 0.870 | 0.330 | 0.074 | >0.9999 | 3.781 | 0.654 | 0.985 | 0.980 |
| Tmem125 | 0.480 | 0.028 | 2.695 | 0.654 | 0.995 | 0.286 | 0.037 | >0.9999 | 1.737 | 0.115 | 0.999 | 1.000 |
| Ugt8a | 0.156 | 0.058 | 0.005 | 0.002 | >0.9999 | 0.105 | 0.014 | >0.9999 | 0.001 | 0.000 | >0.9999 | >0.9999 |
| Xcr1 | 0.028 | 0.006 | 11.489 | 1.331 | 0.600 | 0.255 | 0.022 | >0.9999 | 6.415 | 0.580 | 0.924 | 0.946 |

| **F2** | | | | | | | | | | | | |
| --- | --- | --- | --- | --- | --- | --- | --- | --- | --- | --- | --- | --- |
|  | **A** | | **B** | | | **C** | | | **D** | | | |
|  | **F0 Saline F1 Saline** | | **F0 Saline F1 HDM** | | | **F0 HDM F1 Saline** | | | **F0 HDM F1 HDM** | | | |
| **Gene** | **Mean** | **SEM** | **Mean** | **SEM** | **p value (B vs A)** | **Mean** | **SEM** | **p value (C vs A)** | **Mean** | **SEM** | **p value (D vs C)** | **p value (D vs B)** |
|  |  |  |  |  |  |  |  |  |  |  |  |  |
| Acox2 | 0.391 | 0.218 | 0.003 | 0.002 | >0.9999 | 0.252 | 0.008 | >0.9999 | 0.000 | 0.000 | >0.9999 | >0.9999 |
| Aida | 0.823 | 0.533 | 21.899 | 4.932 | 0.684 | 1.499 | 1.132 | >0.9999 | 9.311 | 2.594 | 0.976 | 0.911 |
| Asmt | 0.453 | 0.114 | 11.931 | 2.237 | 0.931 | 0.657 | 0.135 | >0.9999 | 4.726 | 1.428 | 0.997 | 0.981 |
| Col23a1 | 8.853 | 0.771 | 12.462 | 1.490 | 0.998 | 5.560 | 0.598 | 0.998 | 5.761 | 0.687 | >0.9999 | 0.985 |
| Dpcd | 0.947 | 0.376 | 0.023 | 0.011 | >0.9999 | 0.766 | 0.117 | >0.9999 | 0.009 | 0.007 | >0.9999 | >0.9999 |
| Dpy19l1 | 2.424 | 0.311 | 23.570 | 3.586 | 0.681 | 1.691 | 0.246 | >0.9999 | 24.905 | 2.627 | 0.613 | 1.000 |
| Eef2 | 2.454 | 0.505 | 0.229 | 0.101 | 0.999 | 1.051 | 0.154 | 1.000 | 0.473 | 0.034 | >0.9999 | >0.9999 |
| Erdr1 | 0.243 | 0.090 | 36.045 | 8.595 | 0.237 | 0.157 | 0.033 | >0.9999 | 3.436 | 0.252 | 0.998 | 0.317 |
| Foxi1 | 0.003 | 0.001 | 11.618 | 1.176 | 0.928 | 0.002 | 0.001 | >0.9999 | 1.497 | 0.380 | 1.000 | 0.951 |
| Kdm6a | 1.132 | 0.175 | 0.272 | 0.087 | >0.9999 | 0.407 | 0.059 | >0.9999 | 0.303 | 0.078 | >0.9999 | >0.9999 |
| Lars2 | 0.186 | 0.053 | 21.374 | 4.439 | 0.680 | 0.255 | 0.026 | >0.9999 | 28.872 | 4.042 | 0.435 | 0.979 |
| Luzp2 | 1.767 | 0.211 | 0.766 | 0.131 | >0.9999 | 1.206 | 0.116 | >0.9999 | 0.679 | 0.065 | >0.9999 | >0.9999 |
| Mrs2 | 0.146 | 0.027 | 0.996 | 0.086 | >0.9999 | 0.300 | 0.041 | >0.9999 | 0.725 | 0.098 | >0.9999 | >0.9999 |
| Muc19 | 0.000 | 0.000 | 3.455 | 0.656 | 0.998 | 0.052 | 0.017 | >0.9999 | 4.458 | 1.261 | 0.996 | >0.9999 |
| Nox3 | 0.014 | 0.004 | 10.088 | 0.896 | 0.952 | 0.228 | 0.060 | >0.9999 | 8.979 | 1.137 | 0.967 | >0.9999 |
| Nron | 0.005 | 0.001 | 16.536 | 3.141 | 0.820 | 0.185 | 0.063 | >0.9999 | 36.088 | 5.476 | 0.235 | 0.732 |
| Oasl2 | 1.581 | 0.061 | 11.574 | 0.408 | 0.953 | 1.865 | 0.141 | >0.9999 | 27.930 | 4.043 | 0.518 | 0.825 |
| Ranbp3l | 0.000 | 0.000 | 0.202 | 0.053 | >0.9999 | 0.000 | 0.000 | >0.9999 | 0.284 | 0.038 | >0.9999 | >0.9999 |
| Smpdl3a | 0.364 | 0.069 | 3.244 | 0.409 | 0.999 | 0.263 | 0.066 | >0.9999 | 2.575 | 0.572 | 0.999 | >0.9999 |
| Tmem125 | 2.926 | 0.440 | 12.156 | 1.561 | 0.962 | 1.593 | 0.247 | 1.000 | 9.220 | 0.545 | 0.978 | 0.999 |
| Ugt8a | 0.335 | 0.084 | 0.006 | 0.004 | >0.9999 | 0.309 | 0.029 | >0.9999 | 0.003 | 0.002 | >0.9999 | >0.9999 |
| Xcr1 | 0.049 | 0.006 | 4.162 | 0.445 | 0.996 | 0.045 | 0.009 | >0.9999 | 2.352 | 0.188 | 0.999 | 1.000 |
| **F3** | | | | | | | | | | | | |
|  | **A** | | **B** | | | **C** | | | **D** | | | |
|  | **F0 Saline F1 Saline** | | **F0 Saline F1 HDM** | | | **F0 HDM F1 Saline** | | | **F0 HDM F1 HDM** | | | |
| **Gene** | **Mean** | **SEM** | **Mean** | **SEM** | **p value (B vs A)** | **Mean** | **SEM** | **p value (C vs A)** | **Mean** | **SEM** | **p value (D vs C)** | **p value (D vs B)** |
|  |  |  |  |  |  |  |  |  |  |  |  |  |
| Acox2 | 0.550 | 0.129 | 0.004 | 0.002 | >0.9999 | 0.428 | 0.154 | >0.9999 | 0.011 | 0.001 | >0.9999 | >0.9999 |
| Asmt | 0.248 | 0.045 | 12.842 | 1.989 | 0.809 | 0.477 | 0.098 | >0.9999 | 16.494 | 3.772 | 0.741 | 0.995 |
| Col23a1 | 11.081 | 1.068 | 10.356 | 1.252 | >0.9999 | 18.417 | 1.745 | 0.961 | 14.272 | 0.898 | 0.994 | 0.994 |
| Dpcd | 1.216 | 0.166 | 0.030 | 0.014 | 1.000 | 0.804 | 0.117 | >0.9999 | 0.011 | 0.001 | >0.9999 | >0.9999 |
| Dpy19l1 | 2.928 | 0.245 | 30.027 | 5.010 | 0.222 | 1.262 | 0.318 | 1.000 | 21.219 | 3.757 | 0.586 | 0.936 |
| Eef2 | 3.660 | 0.347 | 0.225 | 0.171 | 0.995 | 2.102 | 0.314 | 1.000 | 0.063 | 0.004 | 0.999 | >0.9999 |
| Erdr1 | 0.490 | 0.124 | 21.594 | 6.804 | 0.441 | 0.384 | 0.111 | >0.9999 | 3.583 | 0.812 | 0.997 | 0.625 |
| Kdm6a | 0.944 | 0.074 | 0.416 | 0.029 | >0.9999 | 0.107 | 0.007 | >0.9999 | 0.969 | 0.133 | >0.9999 | >0.9999 |
| Luzp2 | 3.035 | 0.878 | 0.930 | 0.112 | 0.999 | 1.680 | 0.227 | 1.000 | 0.578 | 0.113 | 1.000 | >0.9999 |
| Mrs2 | 0.331 | 0.059 | 0.572 | 0.091 | >0.9999 | 0.376 | 0.090 | >0.9999 | 0.732 | 0.068 | >0.9999 | >0.9999 |
| Muc19 | 0.041 | 0.013 | 4.297 | 0.760 | 0.991 | 0.020 | 0.005 | >0.9999 | 6.820 | 0.459 | 0.973 | 0.998 |
| Nox3 | 0.764 | 0.064 | 12.339 | 1.178 | 0.845 | 1.640 | 0.334 | >0.9999 | 13.527 | 0.787 | 0.875 | 1.000 |
| Nron | 1.069 | 0.150 | 20.947 | 2.595 | 0.495 | 0.941 | 0.155 | >0.9999 | 18.840 | 2.382 | 0.669 | 0.999 |
| Ranbp3l | 0.026 | 0.002 | 0.421 | 0.059 | >0.9999 | 0.031 | 0.005 | >0.9999 | 0.213 | 0.049 | >0.9999 | >0.9999 |
| Smpdl3a | 0.387 | 0.061 | 2.746 | 0.403 | 0.998 | 0.286 | 0.019 | >0.9999 | 2.263 | 0.409 | 0.999 | >0.9999 |
| Tmem125 | 0.902 | 0.043 | 3.503 | 0.438 | 0.998 | 3.250 | 0.547 | 0.999 | 1.117 | 0.192 | 0.999 | 0.999 |
| Ugt8a | 0.174 | 0.043 | 0.040 | 0.019 | >0.9999 | 0.130 | 0.048 | >0.9999 | 0.039 | 0.010 | >0.9999 | >0.9999 |
| Xcr1 | 0.084 | 0.019 | 8.804 | 0.671 | 0.926 | 0.054 | 0.006 | >0.9999 | 8.996 | 1.019 | 0.942 | >0.9999 |
